# A circadian rhythm-related gene signature for predicting relapse risk and immunotherapeutic effect in prostate adenocarcinoma

**DOI:** 10.18632/aging.204288

**Published:** 2022-09-13

**Authors:** Jin Liu, Zhao Tan, Shijie Yang, Xinda Song, Wenping Li

**Affiliations:** 1 Department of Urology, The Third Hospital of Hebei Medical University, Shijiazhuang, China

**Keywords:** circadian rhythm, immune checkpoint inhibitor, prognosis, prostate adenocarcinoma, FBXL22

## Abstract

Prostate adenocarcinoma (PRAD) represents the most common male carcinoma in developed countries, its high relapse risk contributes to the second-leading cause of cancer-related deaths. Therefore, it is required to develop an effective signature for predicting the relapse risk of PRAD. To identify a circadian rhythm- (CR-) related predictive signature, we analyzed RNA-seq data of patients with prostate adenocarcinoma (PRAD) from the TCGA and GEO cohort. Seven circadian rhythm- (CR-) related genes (*FBXL22*, *MTA1*, *TP53*, *RORC*, *DRD4*, *PPARGC1A*, *ZFHX3*) were eventually identified to develop a CR-related signature. AUCs for 3-year overall survival were 0.852, 0.856 and 0.944 in the training set, validation set and an external independent test set (GSE70768), respectively. Kaplan-Meier curve analysis showed that the high-risk group has a reduced relapse-free survival (RFS) than the low-risk group in the training set, validation set, and test set, respectively (*P* < 0.05). We constructed a prognostic nomogram combining the CR-related signature with T staging to precisely estimate relapse risk of PRAD patients. Finally, we observed that the CR-related gene signature was associated with tumor mutation burden, multiple immune checkpoint molecules and microsatellite instability, and thus could predict response to immune checkpoint inhibitors in PRAD. Conclusively, we developed a circadian rhythm-related gene signature for predicting RFS and immunotherapy efficacy in prostate adenocarcinoma.

## INTRODUCTION

Prostate adenocarcinoma (PRAD) represents the most common male carcinoma in developed countries [1]. In spite of substantial efforts invested into therapeutic development of PRAD, its high relapse risk contributes to the second-leading cause of cancer-related deaths [2–4]. Therefore, it is clinically necessary to identify a useful signature for PRAD to guide cancer treatment. Presently, the most common clinical indicators of cancer relapse are including prostate-specific antigen (PSA), Gleason score, and tumor stage [5, 6]. However, due to the heterogeneity of PRAD, the predictive ability of these common indicators is not unmet. Thereby, to identify a novel biomarker for predicting relapse risk for PRAD is an urgent and relevant effort.

Circadian rhythms are 24-hour oscillations that affect multiple biological functions in humans [7]. Circadian rhythm disorders are linked to aggressive tumor behaviors and unwanted clinical outcomes. Circadian-related genes have been implicated in the pathogenesis of colorectal cancer [7], prostate cancer [8], and bladder cancer [9]. Meanwhile, emerging evidence points to its involvement in tumor microenvironment [10–12]. While circadian rhythm is a hot topic of cancer research recently, the specific mechanisms of its role in humans are unclear. Besides, it is unreported whether circadian rhythm- (CR-) related gene signature can be a prognostic biomarker for PRAD patients.

To establish a CR-related predicting signature for PRAD patients, we investigated bulk RNA sequencing (RNA-seq) profiles from the Cancer Genomic Atlas (TCGA) and Gene Expression Omnibus (GEO), hoping provide an applicable gene signature for predicting prognosis for PRAD patients.

## MATERIALS AND METHODS

### Acquisition of data

Gene expression profiling and clinical information for PRAD patients from TCGA were obtained from UCSC Xena on Sep 9, 2021 (https://xena.ucsc.edu/). Microarray RNA-seq data and survival information of 29 PRAD patients was retrieved from GSE70768 in GEO. GSE70768 was sequenced using the platform of GPL10558 (Illumina HumanHT-12 V4.0 expression beadchip). RNA-seq data from TCGA and GSE70768 were normalized in the form of TPM values and then log2(x + 1) transformed.

Somatic nucleotide variation (SNV) data of PRAD patients were obtained from the TCGA database.

### Estimation of enrichment scores for individual patients

To quantify the expression levels of CR gene set in individual patients, we estimated enrichment score (ES) of the CR-related gene set for individual PRAD patients using single-sample gene set enrichment analysis (ssGSEA) [13]. ssGSEA is a mathematic methodology to estimate relative expression levels of a given gene set using RNA-seq data. The parameters used in this study were as follows: min.sz = 1, max.sz = Inf, tau = 0.25.

### Construction of the gene signature

338 PRAD patients from TCGA were randomly divided into the training set (n = 236) and the validation set (n = 102). The CR-associated genes were screened for eligible genes for establishing the predictive signature using univariate Cox regression firstly, and then further analyzed using least absolute shrinkage and selection operator (LASSO) regression. Then, the eligible genes in LASSO were utilized to construct a gene signature based on eligible genes’ expression levels and their corresponding coefficients in LASSO, using the following formula: FBXL22×(-0.3746637) + MTA1×(0.9070002) + TP53×(-0.2111043) + RORC×(-0.4931651) + DRD4×(0.4730237) + PPARGC1A×(-0.2315328) + ZFHX3×(-0.5221173).

### Assessment of the predictive performance of the gene signature

The predictive ability of the gene signature was assessed mainly by two analyses including receiver operating characteristic (ROC) curve and Kaplan-Meier (KM) curve. Area under the curve (AUC) and log-rank test in were performed in the training set, the validation set, and an independent test set.

### Functional enrichment analysis

Functional enrichment analysis was carried on using the R package “clusterProfiler” (version: 3.18.1) [14]. This toolkit can determine whether canonical biological processes and signaling pathways are significantly enriched in a given patient cohort based on gene expression profiles. The information of canonical biological functions and signaling pathways are available in Bioconductor annotation data GO.db and KEGG.db.

### Construction of a nomogram

To better predict the prognosis of PRAD patients, we conjointly analyzed the gene signature and several common clinical characteristics using multivariate Cox regression analysis and established a predicting nomogram using R package ‘rms’.

### Statistics

Statistical analysis was carried on using R software (Version 4.0.1). Independent sample *t* test or Wilcoxon signed rank test was utilized according to the homogeneity of variance and normal distribution of data. Spearman’s correlation coefficient was performed to investigate the relationship between two continuous variables. Statistical significance was considered when *P* value is less than 0.05.

### Availability of data and materials

All data generated and described in this article are available from the corresponding web servers, and are freely available to any scientist wishing to use them for noncommercial purposes, without breaching participant confidentiality. Further information is available from the corresponding author on reasonable request.

## RESULTS

### Establishment of a circadian rhythm- (CR-) related gene signature

**Figure 1:**
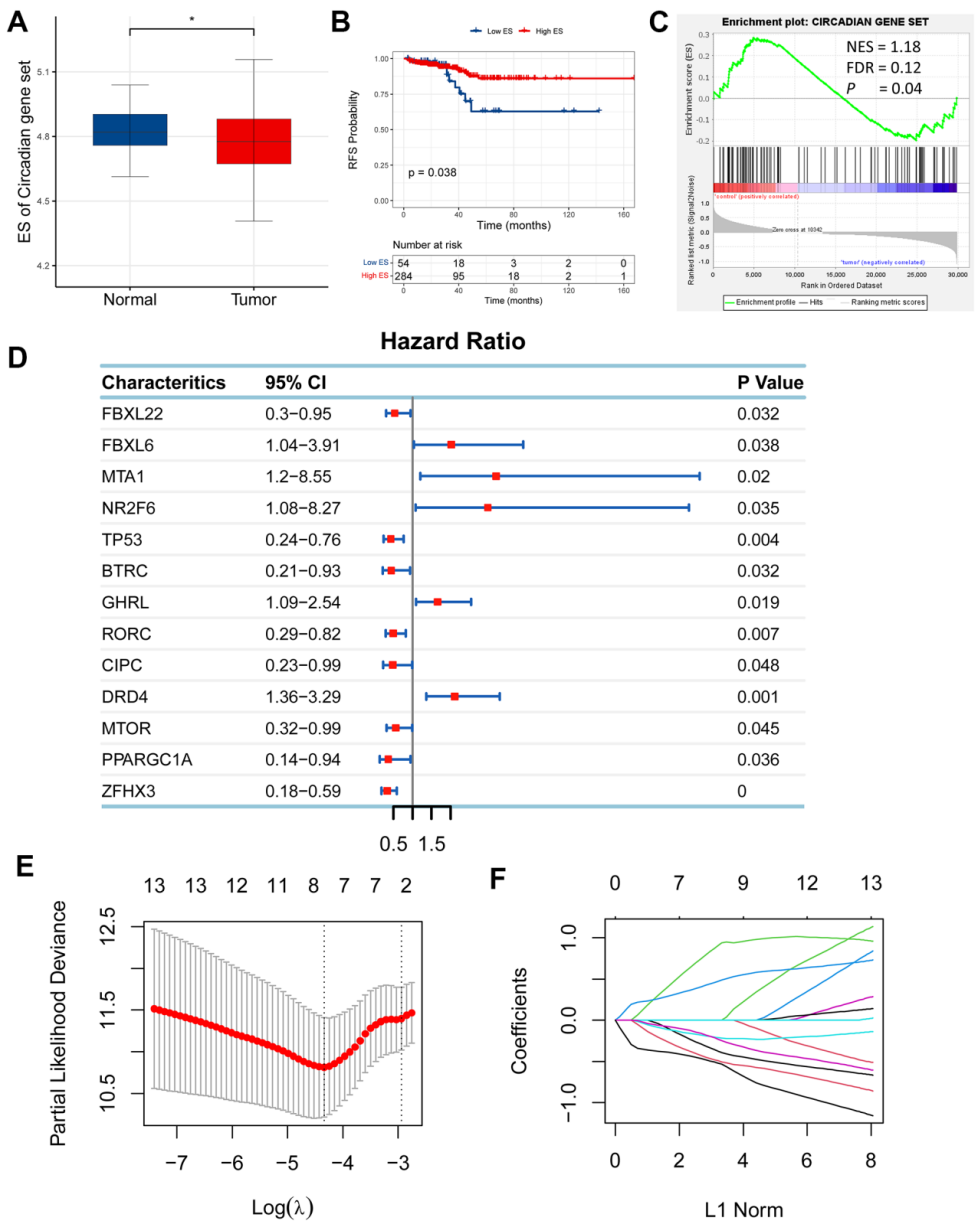
**Establishment of a circadian rhythm- (CR-) related gene signature in prostate adenocarcinoma (PRAD).** (**A**) Enrichment score (ES) of CR-related gene set was significantly enriched in normal tissue than tumor tissue. (**B**) High-ES patients had an improved relapse-free survival (RFS) than their counterparts. (**C**) CR-related gene set was positively enriched in normal tissue compared with tumor tissue. (**D**) 13 CR-related genes were qualified in univariate Cox regression analysis (*FBXL22*, *FBXL6*, *MTA1*, *NR2F6*, *TP53*, *BTRC*, *GHRL*, *RORC*, *CIPC*, *DRD4*, *MTOR*, *PPARGC1A*, *ZFHX3*; *P*<0.05). (**E**, **F**) 13 qualified genes were further filtered using LASSO regression analysis to eliminate multicollinearity and seven eligible genes (*FBXL22*, *MTA1*, *TP53*, *RORC*, *DRD4*, *PPARGC1A*, *ZFHX3*) were eventually acquired for establishment of a CR-related gene signature for predicting RFS in PRAD patients.

Circadian rhythm (CR) is reported to be implicated in cancer [7], whereas it remains unclear whether it has an effect on prostate adenocarcinoma (PRAD). To investigate its association with PRAD, we compared the expression levels of circadian rhythm signaling pathway between PRAD and normal tissues using gene expression profiles of PRAD patients from TCGA. The expression levels of circadian rhythm signaling pathway was quantified as enrichment score (ES) using ssGSEA algorithm based on RNA-seq data of 551 samples from the TCGA cohort of PRAD patients, and the findings showed that ES was significantly increased in normal tissue than in tumor tissue (Figure 1A). Moreover, we divided PRAD patients into the low-ES and high-ES groups according to a median value of ES, and performed a survival analysis. High-ES patients had an improved relapse-free survival (RFS) than low-risk patients (Figure 1B). Gene set enrichment analysis (GSEA) also supported that CR signaling pathway was significantly enriched in normal tissue compared with tumor tissue (Figure 1C). These findings indicated that circadian rhythm had a relationship with PRAD.

Following the finding of the relationship between CR and PRAD, we wondered if CR could predict prognosis for PRAD patients and then planned to develop a circadian rhythm- (CR-) related gene signature to predict survival of PRAD patients. To identify eligible CR-related genes in PRAD, we first performed univariate Cox regression analysis for 117 CR-related genes, and obtained 13 genes (*FBXL22*, *FBXL6*, *MTA1*, *NR2F6*, *TP53*, *BTRC*, *GHRL*, *RORC*, *CIPC*, *DRD4*, *MTOR*, *PPARGC1A*, *ZFHX3*; *P*<0.05; Figure 1D). Then, 13 eligible genes were further filtered using LASSO regression analysis to eliminate multicollinearity and seven eligible genes (*FBXL22*, *MTA1*, *TP53*, *RORC*, *DRD4*, *PPARGC1A*, *ZFHX3*) were eventually acquired for establishment of a CR-related gene signature for predicting RFS in PRAD patients (Figure 1E, 1F). The CR-related gene signature was quantified based on the mRNA expression levels and the corresponding coefficients of seven CR-related genes, using the following formula: FBXL22×(-0.3746637)+MTA1×(0.9070002)+TP53×(-0.2111043)+ RORC×(-0.4931651)+DRD4×(0.4730237)+ PPARGC1A×(-0.2315328)+ ZFHX3×(-0.5221173). The coefficients represented the influence of genes on relapse risk; the positive represented a risk factor for relapse, while the negative represented a protective factor for relapse.

### Assessment of predicting performance of the gene signature

**Figure 2:**
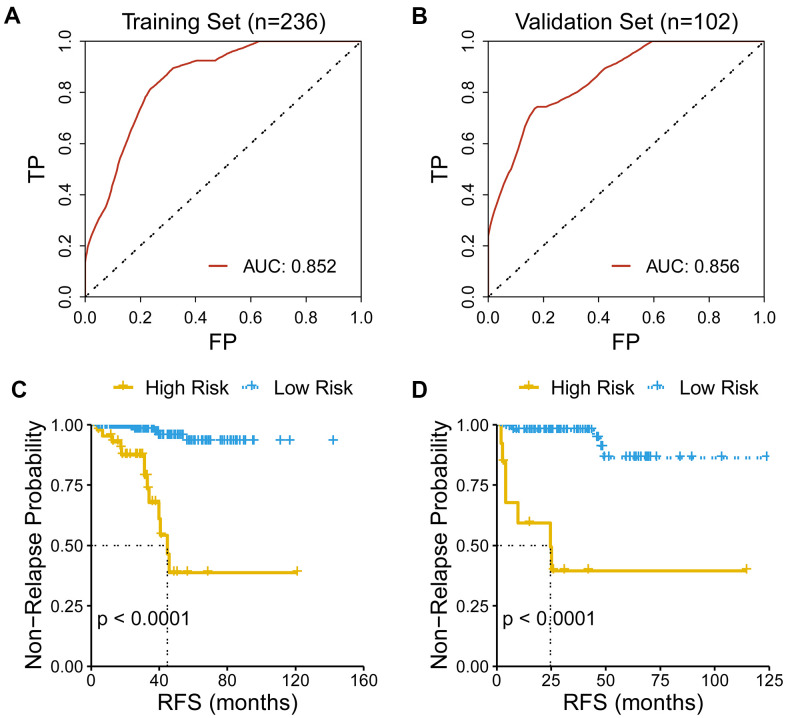
**Evaluation of the performance of the gene signature.** (**A**) AUC for predicting three-year RFS is 0.852 in the training set (n=236). (**B**) AUC for predicting three-year RFS is 0.856 in the validation set (n=102). (**C**) Low-risk patients had an improved RFS than high-risk patients in the training set. (**D**) Low-risk patients had an improved RFS than high-risk patients in the validation set.

The predicting capacity of the CR-related gene signature was validated using ROC curve and KM curve in the training set (n = 236) and the validation set (n = 102). Area under the curve (AUC) for predicting 3-year overall survival were 0.852 and 0.856 in the training set and validation set, respectively (Figures 2A–2B). Then, we quantified the risk of relapse for PRAD patients as risk score using the above mentioned formula, and divided patients into the low- and high-risk patients according to the median value of risk score. Consistent with the above results, survival analysis also revealed that low-risk group had an improved RFS than high-risk group both in the training set and in the validation set (log-rank test, *P* <0.001).

### Discriminative ability of the CR-related signature

**Figure 3:**
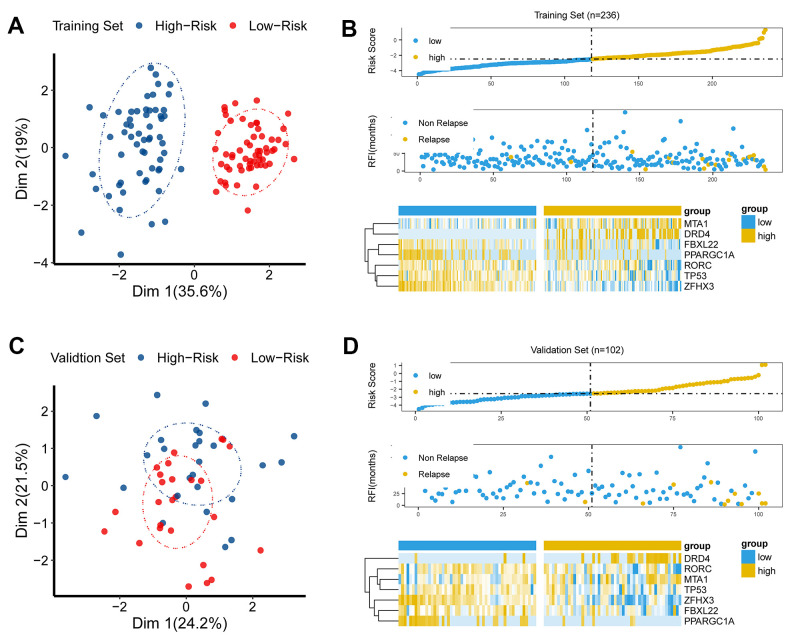
**The discriminative power of the CR-related signature.** (**A**) Principle component analysis (PCA) demonstrated that low-risk group was apparently distinct from high-risk group in Dim 1 in the training set. (**B**) As risk score increased, chance of tumor recurrence increased in the training set. (**C**) Principle component analysis (PCA) showed that the low-risk group was apparently distinct from the high-risk group in Dim 1 in the validation set. (**D**) As risk score increased, chance of tumor recurrence increased in the validation set.

To investigate the discriminative ability of the CR-related signature, we performed principal component analysis (PCA) using seven selected CR-related genes for PRAD patients, and observed a distinction between the low- and high-risk patients (Figure 3A, 3C). Then, we explored the association among gene signature, relapse status and seven gene expression levels. The results demonstrated that risk score was linked to relapse status and the expression of seven genes (Figure 3B, 3D).

### Comparison of predicting performance of the CR-related gene signature with other indicators

**Figure 4:**
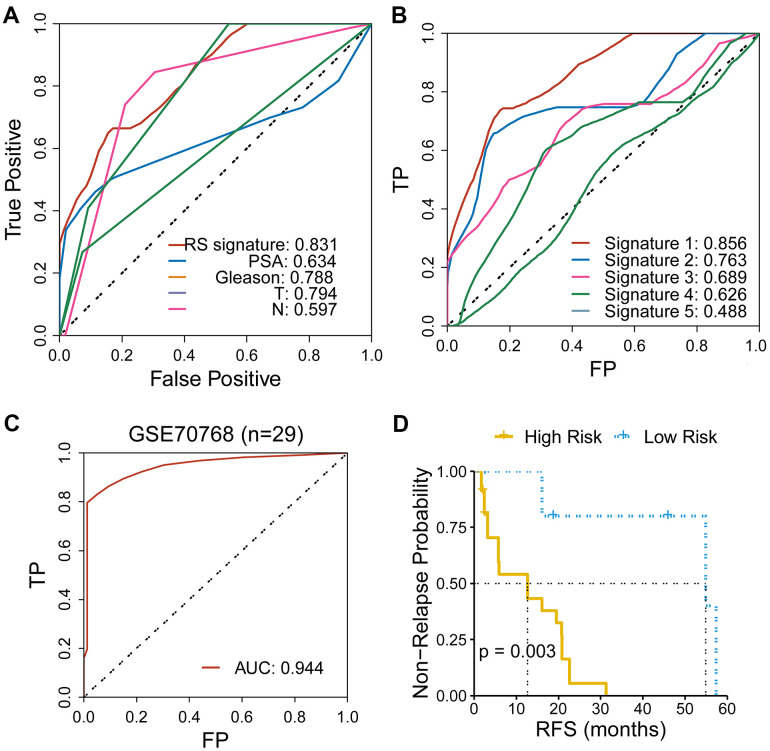
**Comparison of the CR-related gene signature with other indicators for RFS in PRAD.** (**A**) CR-related gene signature showed an improved predictive performance than other clinical indicators, with an AUC of 0.831. (**B**) CR-related gene signature (Signature 1) showed an improved predictive performance than other four reported gene signatures. (**C**) CR-related gene signature also showed an ideal predictive performance in an independent cohort of PRAD (GSE70768), with an AUC of 0.944. (**D**) Low-risk group had an improved RFS than high-risk group in an independent cohort of PRAD (GSE70768) (with a cutoff point of median value; log-rank test, *P* = 0.003).

To further evaluate the predicting performance of the CR-related gene, we compared the predicting capability of the CR-related gene with other indicators of RFS, including clinical characteristics and other four reported gene signatures. The results showed that CR-related gene signature showed an improved predictive performance than other clinical indicators (PSA, Gleason, clinical T stage, clinical N stage), with an AUC of 0.831 (Figure 4A). Meanwhile, CR-related gene signature (Signature 1) also showed an improved predictive performance than other four reported gene signatures: the immune-related gene signature [15] (Signature 2), the metabolism-related gene signature [16] (Signature 3), the PPP1R12A-related gene signature [16] (Signature 4), the TMB-related gene signature [17] (Signature 5), with an AUC of 0.856 VS 0.763, 0.689, 0.626 and 0.488 (Figure 4B).

Furthermore, we assessed the predicting ability of CR-related gene signature in an independent cohort of PRAD patients (GSE70768) using ROC curve. Surprisingly, the CR-related gene signature manifested an impressive predictive ability, with AUC of 0.944 (Figure 4C). Consistently, we also quantified the risk of RFS for patients using the above mentioned formula and divided patients into the low- and high-risk group based on a cutoff point of median value. Low-risk group had a significantly improved RFS than high-risk group (log-rank test, *P* = 0.003; Figure 4D). These results further underlined the predicting capacity of the CR-related gene signature for predicting RFS in PRAD patients.

### Functional enrichment analysis

**Figure 5:**
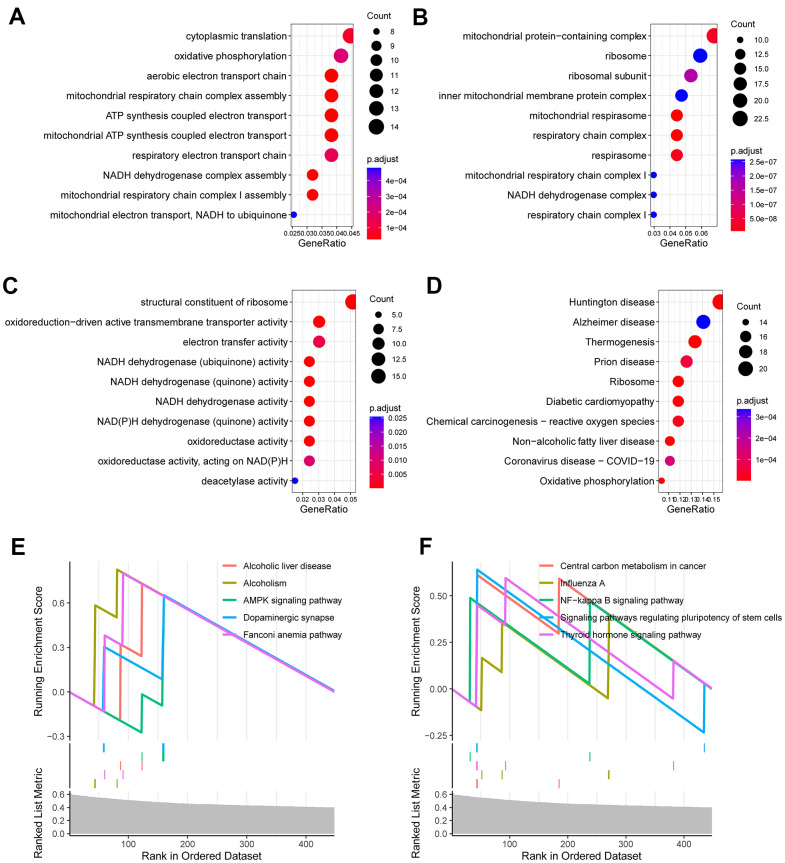
**Functional enrichment analysis for CR-related gene signature.** (**A**) Enriched biological processes included cytoplasmic translation, oxidative phosphorylation, mitochondrial respiratory chain complex assembly. (**B**) Enriched cell components included mitochondrial protein-containing complex, ribosome, inner mitochondrial membrane protein complex. (**C**) Enriched molecular functions included structural constituent of ribosome, oxidoreduction-driven active transmembrane transporter activity and electron transfer activity. (**D**) Enriched KEGG pathways included ribosome, diabetic cardiomyopathy, chemical carcinogenesis (**E**, **F**) Gene set enrichment analysis (GSEA) showed the top 10 KEGG signaling pathway including AMPK signaling pathway, dopaminergic synapse, central carbon metabolism in cancer, NF-kappa B signaling pathway and others.

To interrogate the biological functions that were linked to the CR-related gene signature, we carried out functional enrichment analysis for genes that correlated with the CR -related gene signature. We calculated the correlation coefficients between the gene signature and all genes. 695 genes were selected as the signature-related genes (*P* < 0.01, *R* > 0.4) [18]. Afterwards, these 695 genes were investigated using R package “clusterProfiler”. The results demonstrated that these genes were mainly enriched in cytoplasmic translation, oxidative phosphorylation, mitochondrial respiratory chain complex assembly, oxidoreduction-driven active transmembrane transporter activity and electron transfer activity (Figure 5A–5D). Gene set enrichment analysis (GSEA) displayed that several enriched biological processes were associated with cancer invasion and metastasis, including AMPK signaling pathway, dopaminergic synapse, central carbon metabolism in cancer, NF-kappa B signaling pathway and others (Figure 5E, 5F).

### Establishment of a nomogram

**Figure 6:**
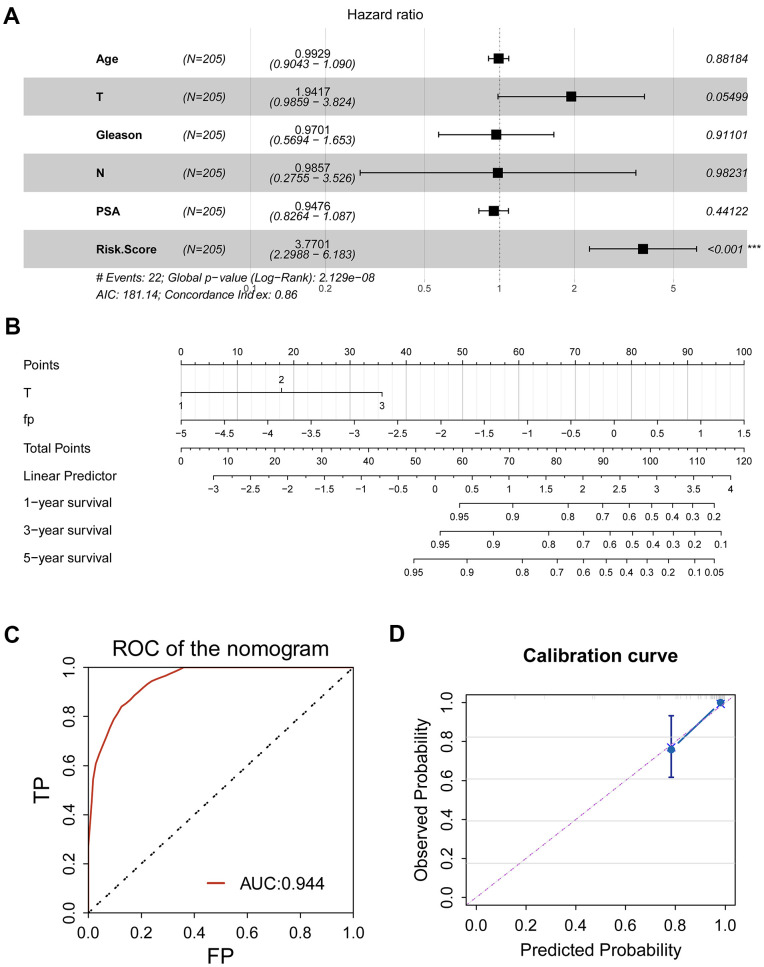
**Clinical application of CR-related gene signature for PRAD patients.** (**A**) Multivariate Cox regression was used to investigate the ability of age, clinical T stage, clinical N stage, Gleason score, prostate specific antigen (PSA) and the CR-related gene signature (risk score) to predict RFS. Risk score remained to be a valid predictor (*P*<0.05). (**B**) A nomogram combing risk score and clinical T stage was constructed to predict 1-, 3- and 5-year RFS for individual PRAD patient. (**C**) ROC analysis for nomogram showed an impressive predictive performance, with AUC of 0.944. (**D**) Calibration curve showed agreement between actual and predicted RFS, indicating an ideal predictive capability.

For better prediction of the survival of the PRAD patients, we endeavored to combine the signature with clinical features to establish a prognostic nomogram. We first analyzed the ability of age, clinical T stage, clinical N stage, Gleason score, prostate specific antigen (PSA) and the CR-related gene signature (risk score) to predict RFS using multivariate Cox regression. The results demonstrated that the gene signature and clinical T stage remained to be a valid predictor (Figure 6A, *P≤*0.05).

Then, we established a prognostic nomogram consisting of the gene signature and pathological T (Figure 6B). To assess prognostic ability of the nomogram, we performed a ROC curve and calibration curve. ROC curve showed an amazing predictive capability, with 5-year AUC of 0.944; consistently, C-index of the nomogram was 0.75±0.05, indicating the discriminatory ability of the nomogram; calibration curve also displayed a consistency between predicted probability and observed probability, suggesting the effectiveness and robustness of the established nomogram.

### Exploration of the association of CR-related gene signature with tumor immune microenvironment

**Figure 7:**
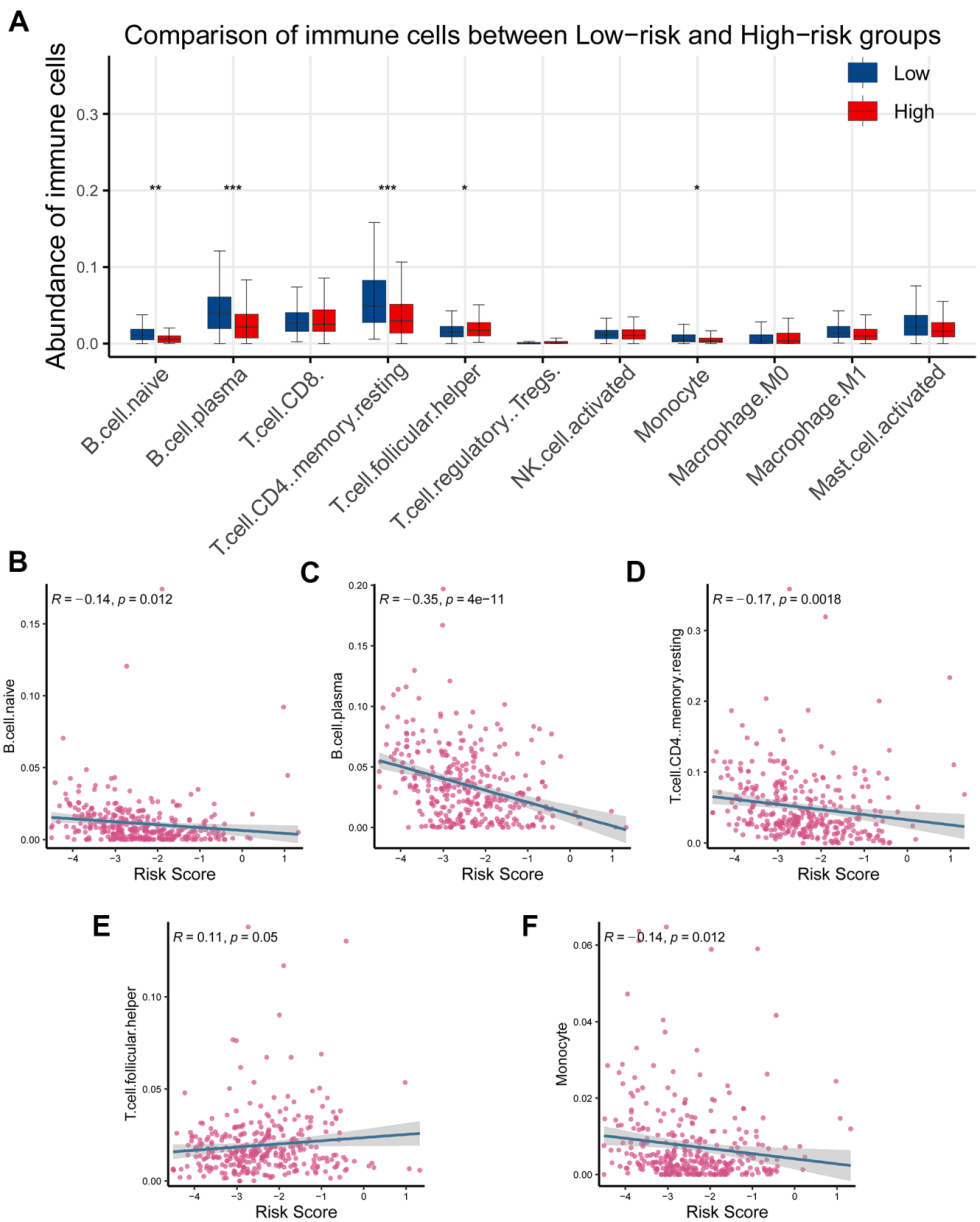
**Association of the CR-related gene signature with tumor infiltrating immune cells in PRAD.** (**A**) Comparison of tumor infiltrating immune cells between low- and high-risk groups demonstrated that there existed a significant difference in the abundance of B cell naive, B cell plasm, T cell CD4 memory, T cell follicular helper, and monocyte (*P* < 0.05). (**B**–**D**) Risk score was inversely correlated with B cell naive, B cell plasm, T cell CD4 memory, T cell follicular helper, and monocyte (*R* < 0, *P* < 0.05). (**E**) Risk score was positively correlated with T cell CD4 memory (*R* < 0, *P* < 0.05). (**F**) Risk score was inversely correlated with monocyte (*R* < 0, *P* < 0.05).

Since CR-related gene signature could predict RFS in PRAD, we next wondered to explore its relationship with tumor immune microenvironment. Tumor immune microenvironment has been reported to be implicated in identification of pivotal molecules, drug response, and novel therapeutic methods. To interrogate the role of CR-related gene signature in PRAD, we computed the proportion of immune cells in cancerous tissue using CYBERSORT and investigated the association between risk score and immune cells. The results showed that T cell follicular helper was significantly elevated, while B cell naive, B cell plasma, T cell CD4 memory resting and monocyte were reduced in high-risk group than low- risk group (*P* < 0.05; Figure 7A). Further correlation analysis supported that T cell follicular helper was positively correlated with gene signature (Figure 7E), while B cell naive, B cell plasma, T cell CD4 memory resting and monocyte were negatively correlated with gene signature (Figure 7B–7D, 7F).

### The profiling of somatic nucleotide variation for PRAD patients between the low- and high-risk patients

**Figure 8:**
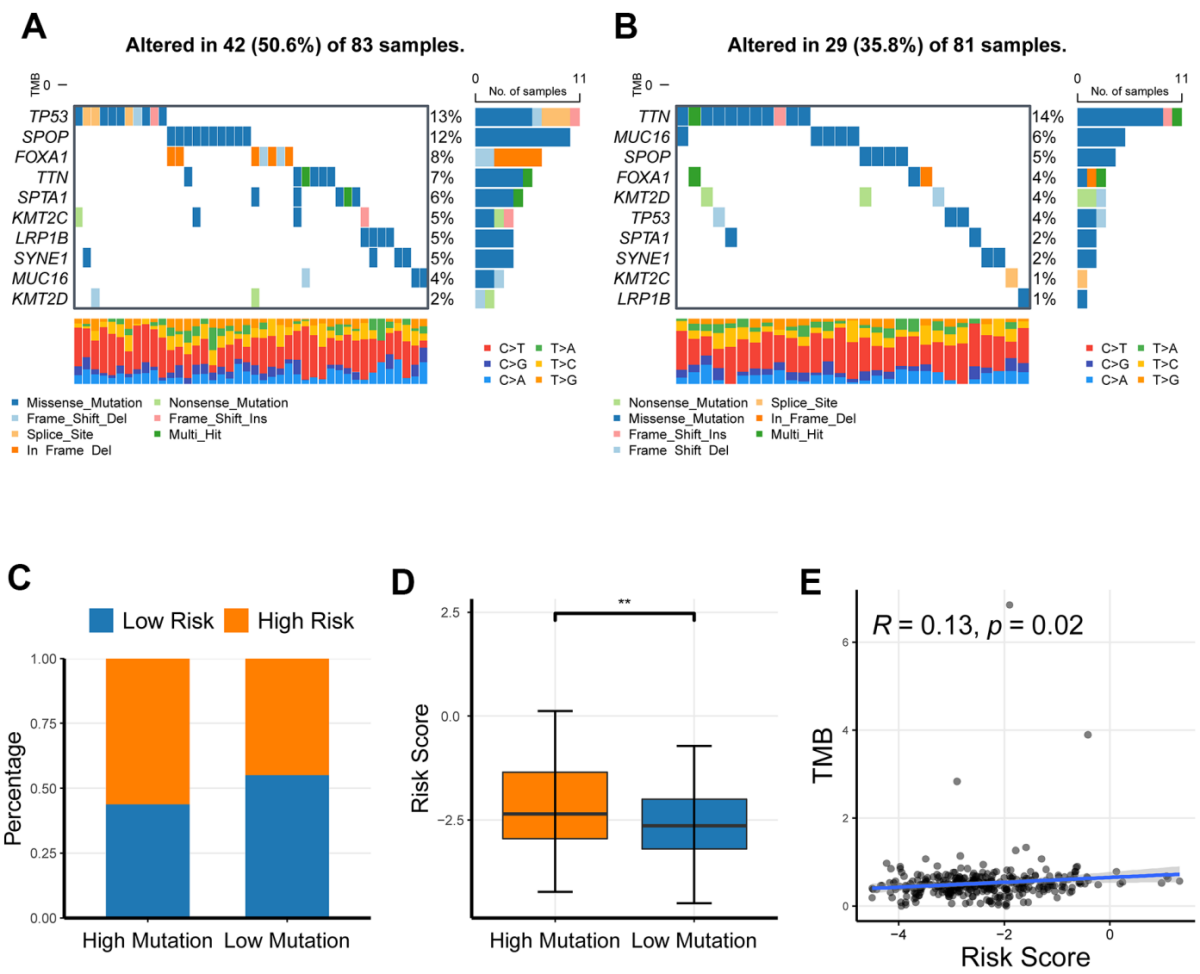
**The profiling of somatic nucleotide variation for PRAD patients of different risk groups.** (**A**, **B**) The waterfall plot of nucleotide variation rate in high-risk group and low-risk group. (**C**, **D**) The bar plot and box plot analyses of risk scores in the high-mutation group and low-mutation group. (**E**) The correlation of tumor mutation burden and risk score. *P <0.05, ** *P* <0.01, ***P<0.001.

To investigate the association of CR-related gene signature with the mutation levels in PRAD patients, we profiled the mutation landscape of the low-risk and high-risk groups, respectively, by analyzing somatic nucleotide variation data of PRAD patients from the TCGA cohort using the maftool R package. To better compare the potential distinction of the mutation landscape between different risk-score groups, we here defined the patients with the top 25% of risk score as the high-risk group and defined the patients with bottom 25% of risk score as the low-risk group. The waterfall plot demonstrated that the high-risk group had a higher nucleotide variation rate than the low-risk group (50.6% vs 35.8%, Figure 8A, 8B). Consistent with the findings of the waterfall plot, the bar plot and box plot analyses also showed that the risk scores were critically increased in the high-mutation group than in the low-mutation group (Figure 8C, 8D). A high somatic nucleotide variation rate has been reported to be associated with tumor mutation burden (TMB) that is reflective of effective response to immune checkpoint inhibitors (ICIs) [19, 20]; Thus, we asked whether the risk score had a relationship with TMB. We first quantified tumor mutation burden (TMB) using the maftool R package based on somatic nucleotide variation data of PRAD patients, and found that TMB was significantly correlated with risk score (*P* = 0.02, Figure 8E). Collectively, these results showed that the risk score could reflect the genomic instability and the TMB level, indicating its potential capability to predict response to ICIs in PRAD patients.

### The effects of CR-related gene signature on response to ICIs

**Figure 9:**
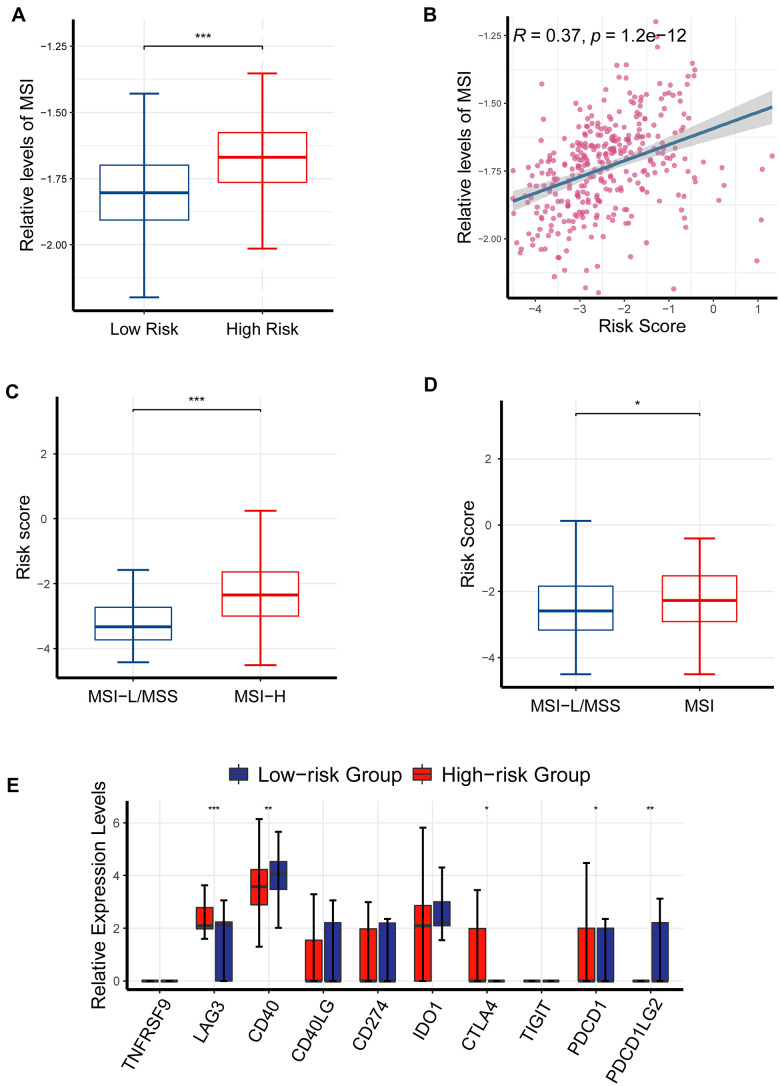
**The effects of CR-related risk score on response to ICIs.** (**A**) MSI levels was elevated in the high-risk patients than in low-risk patients (*P* <0.001). (**B**) MSI levels were positively correlated with risk score (*P* < 0.05, *R* = 0.37). (**C**) Risk score was significantly increased in MSI-H group than MSI-L/MSS group (data of MSI generated from PreMSIm R package; *P* < 0.001). (**D**) Risk score was significantly higher in MSI-H group than MSI-L/MSS group (data of MSI generated from UCSCXenaShiny R package; *P* < 0.05). (**E**) Comparison of multiple immune checkpoint mRNA (*PDCD1*, *LAG3*, *CD40*, *CTLA4*, *PDCD1LG2*) in the high-risk patients and the low-risk patients. *P <0.05, ** *P* <0.01, ***P<0.001.

Elevated TMB, high-microsatellite instability (MSI) and immune checkpoint molecules have been demonstrated to be predictive of response to immune checkpoint inhibitors (ICIs) in cancer patients [21]. The previous results had suggested that CR-related risk score had a significantly impact on RFS, genomic stability and TMB; thereby we asked if it was also associated with MSI status and immune checkpoint molecules in PRAD. To enhance the reliability of results, we quantified MSI levels using three different strategies, including ssGSEA, PreMSIm and UCSCXenaShiny. MSI levels was significantly elevated in high-risk group than in low-risk group (*P* <0.001; Figure 9A), and positively correlated with risk score (*P* < 0.05, *R* = 0.37; Figure 9B). Consistently, risk score was also significantly higher in MSI-H group than MSI-L/MSS group using PreMSIm method (Figure 9C; *P* <0.001). Meanwhile, risk score was significantly higher in MSI-H group than MSI-L/MSS group using UCSCXenaShiny method (*P* <0.05; Figure 9D).

Consistently, the expression levels of *LAG3*, *PDCD1* and *CTLA4* were also critically upregulated in the high-risk patients than in the low-risk patients (Figure 9A), underlining the ability of CR-related gene signature to predict drug response (Figure 9E). Collectively, these findings further underlined the ability of CR-related gene signature to predict immunotherapeutic response in PRAD.

## DISCUSSION

We established a seven-gene CR-related signature for predicting relapse risk in PRAD patients, developed a nomogram consisting of the CR-related gene signature and T staging. Several enriched biological processes were related to AMPK signaling pathway, dopaminergic synapse, central carbon metabolism in cancer, and NF-kappa B signaling pathway. Moreover, we observed that the CR-related gene signature was associated with tumor mutation burden, multiple immune checkpoint molecules and microsatellite instability, and thus could predict response to immune checkpoint inhibitors in PRAD. Overall, this study provided a novel predictor of relapse risk and immunotherapeutic effects of patients with PRAD, which would help to develop precision medicine.

One contribution of this research is the development of a CR-related gene signature for predicting clinical outcomes of PRAD patients. The predicting ability of the signature has been validated in an independent cohort (GSE70768) and compared with common clinical index and some reported gene signatures to prove the superiority of its predicting ability.

Another important finding that several important biological processes were identified to be involved in CR and high relapse risk, including AMPK signaling pathway, dopaminergic synapse, central carbon metabolism in cancer, and NF-kappa B signaling pathway. Consistent with our findings, AMPK signaling pathway has been reported to involve in PRAD [22–24], and targeting AMPK signaling pathway with CO or metformin can suppress prostate cancer cell growth [25, 26]. Remarkably, it is unreported that whether dopaminergic synapse is related to PRAD. Here, we observed the association between dopaminergic synapse and PRAD, which requires further investigation. Central carbon metabolism has a key role in metabolic programming [27], and regulation of carbon metabolism is essential for the treatment of prostate cancer. Current studies have pointed out that NF-kappa B signaling pathway is involved in castration-resistant prostate cancer [28, 29] and associated with prostate cancer cell EMT and bone metastasis [30, 31]. Here, we found CR-related gene signature was associated with these oncogenic signaling pathways, suggesting the role of circadian rhythm disruption in the development of cancer.

Moreover, the seven genes consisting of the CR-related gene signature could be potential biomarkers for the development and relapse risk of prostate cancer. *FBXL22* has been identified to correlated with ER- breast cancer in an exome-wide analysis [32]; nevertheless, its specific effects on cancer, including prostate cancer, is unclear. Here, the present study indicated its role in predicting recurrence risk of prostate cancer. *MTA1* has been reported to drive malignant progression and bone metastasis in prostate cancer in several studies [33, 34], whereas the protective effect of TP53 is well-known [35, 36]. *RORC* encoding ROR-γ could induce androgen expression in prostate cancer [37] and is a potential therapeutic target in castration-resistant prostate cancer [38]. *PPARGC1A* and *ZFHX3* are also reported to be associated with prostate cancer [39, 40], which could partly explain their ability to predict relapse risk.

This research has several practical meanings for clinical management of PRAD patients. Firstly, we developed a new gene signature and a novel predicting nomogram that would benefit therapeutic strategies for PRAD. In addition, we identified multiple pivotal genes and signaling pathways that would help development of new therapeutic strategies for PRAD. Needless to say, this study was mainly performed using bioinformatics, and thus laboratory experiments are needed for further research.

The present study has several limitations that need attention. Firstly, the pivotal gene and signaling pathways in this study were identified using bioinformatics analysis, laboratory experiments are needed to investigate their biological rationales. In addition, the risk score was identified to predict response to immunotherapy of PRAD, warranting further clinical investigation.

Conclusively, we successfully developed a new CR-related signature and a nomogram that could predict relapse risk of PRAD patients, which could provide insights into further researches on PRAD.
